# Use of oral retinoids in the perioperative period by surgical specialty: a literature review

**DOI:** 10.1007/s00403-024-03750-2

**Published:** 2025-01-06

**Authors:** Lauren Truax, Morgann Hendrixson, Jaree Naqvi, Julian Trevino

**Affiliations:** 1 Department of Surgery, Wright State University Boonshoft School of Medicine, 30 E. Apple St. Suite 6258, Dayton, OH 45409 USA; 2 Wright State University Boonshoft School of Medicine, Dayton, OH USA; 3 Department of Dermatology, Wright State University Boonshoft School of Medicine, Dayton, OH USA

**Keywords:** Perioperative retinoids, Surgery, Isotretinoin, Oral retinoids, Wound healing

## Abstract

This review examines the impact of oral retinoids, particularly isotretinoin, on incisional wound healing across surgical specialties. Commonly prescribed for dermatologic conditions, concerns persist regarding oral retinoids’ potential adverse effects on wound healing, prompting the widespread practice of discontinuing these medications before surgery. We performed a PubMed search and analyzed research published regarding the use of oral retinoids in a variety of surgical subspecialties: dermatologic, plastic, ophthalmologic, orthopedic, ENT/otologic, and maxillofacial. Contrary to conventional practices, our review challenges the necessity of discontinuing oral retinoids before surgery in many instances based currently available research. Our literature review underscores the need for individualized risk-benefit assessment when deciding whether to hold retinoid therapy prior to surgical intervention as well as the need for more data surrounding systemic retinoids and wound healing interactions. While encouraging a reevaluation of current practices, we advocate for evidence-based decision-making in perioperative care across various surgical specialties in collaboration between dermatologists and surgeons.

## Introduction

Oral retinoids are prescribed to treat numerous dermatologic conditions. The most commonly used systemic retinoid is isotretinoin in the treatment of acne in adolescents and young adults. While the indications for the use of oral retinoids are well-established, the issue of stopping their use prior to surgery remains controversial. Postponing elective surgery until cessation of oral retinoids, or at the very least pausing use of oral retinoids in the perioperative period, is a common clinical practice. This practice is based on the belief that oral retinoids negatively impact wound healing and increase the risk of undesirable outcomes such as hypertrophic scarring and keloid formation [1–4]. Case studies published in the 1980s reported a causal association between the use of oral retinoids and adverse wound healing. Keloid formation was reported in a combined 9 patients who underwent argon laser or dermabrasion treatments while taking isotretinoin [5, 6]. Mechanisms have since been proposed to explain the phenomenon and describe how isotretinoin may increase the risk of poor wound healing. It is known to physiologically inhibit sebaceous gland activity which is one of the structures from which epithelial cells regenerate [1, 7, 8]. Additionally, isotretinoin may suppress collagenase leading to collagen buildup and, theoretically, keloid formation [9]. However, in subsequent decades, little research has been conducted to further evaluate the effect, if any, oral retinoids have on wound healing in the clinical setting. Furthermore, it is unclear if this effect is isolated to isotretinoin or occurs generally with the oral retinoid class. The little existing research calls into question whether oral retinoids truly adversely affect wound healing. In this literature review, we report existing data on this topic and whether the current practice of holding retinoids or postponing surgery is supported scientifically. We also sought to determine whether any surgical societies have published guidelines to determine if, and when, oral retinoids should be discontinued in the perioperative period. We further parsed our findings by surgical specialty to determine how practice varies amongst different specialists.

## Methodology

We conducted searches of the PubMed database using search terms “retinoids,” “isotretinoin”, “surgery”, “procedure”, “wound healing”, “keloid”, “laser”, and included manuscripts from inception to November 1, 2023. Articles returned were filtered based on title and abstract reading. Literature deemed relevant for this project focused on procedures and surgeries performed in patients who received concurrent oral retinoids or retinoids within 6 months of the surgical intervention. Those articles considered relevant were fully read. Articles that were not written in the English language were excluded. We focused primarily on wound healing outcomes in patients who received perioperative retinoids.

## Findings

### Dermatologic procedures/surgeries

Oral retinoids are most commonly prescribed by dermatologists, so the majority of literature regarding perioperative use of retinoids is found within the field of dermatology. Recently, an American Society for Dermatologic Surgery taskforce created a consensus statement regarding the safety of isotretinoin use in the perioperative period. These consensus recommendations were created after an in-depth literature search of perioperative (within 6 months) isotretinoin use and subsequent scarring following a variety of dermatologic surgeries/procedures. Their consensus was stated as follows:

“…There is insufficient evidence to justify delaying treatment with superficial chemical peels and nonablative lasers, including hair removal lasers and lights, vascular lasers, and nonablative fractional devices for patients currently or recently exposed to isotretinoin. Superficial and focal dermabrasion may also be safe when performed by a well-trained clinician.” [10].

In light of ongoing research on the safety of perioperative retinoids, the remainder of this section will focus on studies of concomitant isotretinoin use with dermatologic procedures that we found to be most relevant given this consensus statement. Chemical peels are commonly utilized in the treatment of acne, photoaging, and pigmentation disorders. In a previous study of 120 patients undergoing superficial chemical peels and other rejuvenation procedures for photoaging, patients who were treated with concomitant low-dose isotretinoin had statistically significant improvement in several skin measures including pigmentation, elasticity, tone, and pore size compared to individuals who did not receive isotretinoin. The only adverse effect reported with concurrent isotretinoin use and chemical peel was lip dryness, reported by less than 10% of participants. There was no evidence of impaired skin healing or keloid scarring in any participants [11]. Similarly, in a study of 60 patients from India receiving biweekly salicylic acid peels for treatment of acne, concomitant use of isotretinoin was associated with greater reduction in the number of acne lesions than isotretinoin alone with no reported issues of skin healing [12]. 

Dermabrasion is commonly used in dermatology to help improve the appearance of facial scarring. Initial studies regarding isotretinoin use and post-dermabrasion skin healing noted atypical keloid formation in 11 total patients [4–6, 13]. More recently however, complete re-epithelialization without any post-procedural scarring was noted in a study of 7 patients (5 Caucasian, 2 African American) treated with less-invasive dermabrasion while on isotretinoin and in a study of 10 patients (8 Caucasian, 1 African American, 1 Asian) who underwent medium-depth chemical peels followed by sandpaper dermabrasion [14, 15]. 

Laser procedures have been used in the field of dermatology since the 1960s for a variety of conditions such as acne, rosacea, and laser hair removal. In the early 1980s, isotretinoin was FDA-approved for treatment of severe acne and off-label uses for conditions such as rosacea and folliculitis commenced [16]. With the goal of optimizing outcomes, some dermatologists began implementing treatment plans involving both laser treatments and isotretinoin. A recent systematic review found that among 871 total patients treated with laser and isotretinoin, there were 12 patients with transient, self-resolving skin reactions, and only 2 patients noted to have keloid scarring [17]. One patient was found to have a separate, unrelated keloid on the knee from a prior procedure [6]. The second patient was an adolescent Asian female who underwent pulsed-dye laser (PDL) treatment for capillary malformations of the chest and face while taking isotretinoin and developed keloidal scarring on the chest [18]. This was the only study found where a patient was treated for a vascular malformation while taking isotretinoin. However, keloid formation has also been documented following treatment of a vascular malformation in a patient not taking isotretinoin [19]. It is important to consider that this patient did have several well-studied risk factors for keloid scarring including her adolescent age and Asian race [20–22]. In addition, this patient developed keloid scarring on her chest, which is the most common site for keloid formation [23]. Based on our literature search, it is unclear if there is increased incidence of hypertrophic or keloidal scarring following PDL treatment of vascular malformations. In summary, the risk of abnormal scarring from laser procedures in individuals with perioperative isotretinoin seems to be low, with most reported cases of abnormal scarring being noted in individuals with known risk factors. These findings emphasize the importance of dermatologists taking a thorough history focused on identifying potential risk factors for abnormal scarring in patients considering laser procedures while taking isotretinoin.

Following these 2 initial studies, a variety of papers were published regarding ablative and non-ablative laser treatments with concomitant isotretinoin use without evidence of keloid or abnormal scarring [24–29]. In addition, no adverse skin healing or scarring was noted in a variety of laser hair removal studies in patients concomitantly taking isotretinoin [30–35]. More recently, a study comparing treatment of acne with isotretinoin alone versus low-dose isotretinoin in combination with pulsed-dye laser noted greater improvement in acne with the combined therapy. There was no evidence of abnormal scarring in either group suggesting potential utility of dual therapy with isotretinoin and pulsed-dye laser in optimizing acne treatment outcomes [36]. Consistent with the findings of the systematic review by Mirza et al. mentioned above, there is insufficient evidence of a causal relationship between perioperative isotretinoin use with laser procedures and abnormal scarring, thus challenging previous beliefs that laser procedures should be delayed in the setting of recent or current isotretinoin use.

Although most of the research regarding the impact of isotretinoin use on wound healing in the setting of dermatologic procedures focuses on chemical peels, dermabrasion, and laser procedures, several other procedures have been noted in the literature. Of note is a case study of surgical debulking of genital warts in combination with isotretinoin. This study found that this combined therapy resulted in complete remission of refractory genital warts in an individual with systemic lupus erythematosus without evidence of abnormal scarring [37]. Additionally, appropriate healing without complication was noted in an individual who underwent several wide excisions of hidradenitis suppurativa lesions in the setting of isotretinoin therapy for the preceding 2 years [38]. While the majority of research on perioperative retinoids focuses on isotretinoin, our review of the literature revealed a study focused on another oral retinoid, acitretin. A second-generation retinoid, acitretin was studied in organ-transplant recipients following excisional surgery or Mohs micrographic surgery for skin cancer. Wound healing was assessed within the first month and then again before the eighth month postoperatively. There was no significant difference in the incidence of hypertrophic granulation tissue, scarring, infection, or dehiscence in the surgical sites of patients treated with acitretin compared to the control group [39]. 

### Plastic surgery

Though aesthetically pleasing outcomes are desirable in all incisional surgeries, they are of emphasized importance in cosmetic plastic surgery. For purposes of our literature review, adolescent and young adult age groups are generally healthy, making finding data on the use of oral retinoids in the setting of incisional surgery a challenge. Breast reduction surgery, however, provides an opportunity to look for incidences of oral retinoid use in the perioperative period. In 2020, the American Society of Plastic Surgery reported that approximately 22% of aesthetic breast reductions performed were on patients aged 13–29 [40]. This makes for an ideal population to search for effects of oral retinoids on wound healing. In their case series, Davies et al. described 2 adolescent female patients who underwent elective breast reduction while taking therapeutic doses of Roaccutane [41]. Both patients continued Roaccutane throughout the perioperative period and for months postoperatively. Incisional breast surgery carries a propensity for hypertrophic scarring, and 1 of the patients had “a small amount of hypertrophic scarring… which settled within the anticipated normal period” and both patients were pleased with their overall outcome in appearance [41]. It is important to note that neither patient had a family or personal history of abnormal scarring. To our knowledge, this is the only observational study commenting on healing after breast surgery in the setting of concurrent oral retinoid use. Importantly, Davies makes a point that postponing surgery until cessation of Roaccutane in the presented patients could have led to negative psychological outcomes. Performing these surgeries in a timely fashion allowed patients to have increased self-esteem and less pain associated with breast size disproportionate to their body habitus. Similarly, another literature review considering transmasculine patients undergoing chest masculinization surgery evaluated the implication of perioperative isotretinoin [42]. They were unable to find any studies examining this scenario, but concluded “in cases of severe acne with a risk of scarring, it is not recommended to… discontinue isotretinoin before chest masculinization surgery,” since many transmasculine patients are prescribed ongoing isotretinoin therapy to combat acne associated with masculinizing hormone treatment [42]. Again, the psychological benefits of breast surgery, which in this scenario falls under gender-affirming care, likely should not be delayed in order to stop oral retinoids.

Plastic and reconstructive surgeons frequently utilize muscle flaps for a wide variety of large anatomic defects (e.g., for reconstruction after mastectomy). Elevated creatine phosphokinase (CPK) is a known side effect of oral isotretinoin, though rhabdomyolysis is a rarely reported complication [43]. As such, there is a theoretical possibility that a patient on systemic isotretinoin treatment may have a muscle flap harvested for reconstruction with pre-existing cellular damage, raising the risk for flap necrosis and failure [1]. There are no reports citing isotretinoin compromising muscle flaps to our knowledge, though in this particular instance if the procedure is elective it would be reasonable to check the patient’s CPK prior to going to the operating room.

Another common plastic surgery procedure that is performed in younger individuals is rhinoplasty, providing another unique opportunity to study perioperative retinoids. A retrospective case series describes 3 patients who received postoperative isotretinoin following rhinoplasty and subsequently developed nasal tip deformities. These deformities included composite graft cartilage prominence in 1 patient, bossae formation in 1 patient, and nasal tip asymmetry in 1 patient. Isotretinoin was initiated 7 months, 1 year, and 2 years after rhinoplasty respectively with deformities arising within the first 6 months of isotretinoin therapy for all 3 patients [44]. Given the extended period of time between the surgeries and isotretinoin initiation, as well as the retrospective nature of the study, it is difficult to establish causation. In a larger prospective study, 350 individuals undergoing rhinoplasty were split into an experimental and a control group. The experimental group began oral isotretinoin 2 weeks prior to rhinoplasty and continued it until 2 months after surgery. Patients were then evaluated 1, 2, 6, and 12 months postoperatively to determine if there were differences between groups with regards to healing. No evidence of abnormal skin healing or scarring was noted in patients in either group, and interestingly, patients who received isotretinoin reported higher satisfaction rates with their surgical outcomes [45]. In addition, a randomized clinical trial published in 2018 sought to understand how isotretinoin affects postoperative cosmetic outcomes in individuals undergoing rhinoplasty. This study randomized 48 individuals determined by expert dermatologists to have “thick skin” into an experimental group to receive postoperative isotretinoin and a control group to receive a placebo. No evidence of abnormal scarring was noted and there were higher rates of satisfaction at 3 and 6 months postoperatively in individuals who received isotretinoin compared to the placebo group. In fact, when cosmetic surgical outcomes were compared between the 2 groups, an expert surgeon who was not involved in the surgeries and was blinded to the patient groups rated those in the group receiving isotretinoin to be superior at both 3 and 6 months postoperatively. This finding was statistically significant. These findings suggest that perioperative use of oral retinoids in patients undergoing rhinoplasty may improve patient satisfaction and aesthetic outcomes [46]. However, the risk of nasal tip deformities cannot be excluded due to the limited but reported outcomes previously described. Therefore, plastic surgeons and dermatologists should discuss the potential benefits and adverse effects with their patients desiring rhinoplasty to arrive at a patient-centered decision on whether to consider isotretinoin use.

### Ophthalmologic surgery

Oral retinoids are reported to have various ophthalmologic side effects, including dry eye and meibomian gland dysfunction (MGD) [47]. As a result, it is often recommended that patients undergoing refractive surgery not be actively taking them. The corneal epithelium of the eye is damaged in laser-assisted in situ keratomileusis (LASIK) surgery. Isotretinoin could negatively impact healing of the cornea due to retinoids altering gene expression in the epithelium of meibomian glands and inhibit meibocyte proliferation, among other mechanisms, leading to their reported side effects of dry eye and MGD [48]. Dry eye and MGD additionally are a well-known complication of LASIK that can compromise flap-healing and visual acuity outcomes, although this is mainly hypothesized to be due to denervation of the cornea and a decreased blink rate, not the same mechanism which retinoids may contribute to MGD [49]. That said, if oral retinoids further increased risk of MGD, it would follow that they may need to be avoided to optimize wound healing after refractive surgery. A recent retrospective cohort study compared outcomes in eyes of patients undergoing refractive surgery (LASIK or photorefractive keratectomy) who were taking isotretinoin at the time of surgery versus those who were not, and failed to find statistically significant differences in visual outcomes or complications [50]. Moreover, a recent retrospective analysis found no difference in outcomes in patients who underwent LASIK surgery with preoperative asymptomatic MGD versus those without [51]. This limited evidence suggests that if an adolescent patient is currently taking isotretinoin to treat their acne and also planning on undergoing LASIK surgery, they may not necessarily need to stop the isotretinoin even if they have MGD so long as it is not symptomatic. Given no clear guidelines exist, caution should still be exercised and an ophthalmologist should be consulted for additional clinical judgment.

### Orthopedic surgery

Orthopedic surgeons, especially those who focus on treating athletes, likely have overlap in patient population with dermatologists treating acne. Wootton et al. assessed whether or not patients currently receiving isotretinoin need to pause therapy prior to surgical repair of sports injuries, specifically, arthroscopic repair of joints [9]. Their review of the current literature failed to find any orthopedic-specific studies that noted concurrent isotretinoin use. In their case study, joint decision making between orthopedic surgeon, dermatologist, and patient led to ultimately holding isotretinoin in the perioperative period, though they do not elaborate how many weeks prior to and after surgery it was held [9]. There were no evident complications. Although this paper focuses on the interaction between oral retinoids and wound healing across surgical specialties, there is little literature discussing wound healing in orthopedic surgery while on oral retinoids. However, research does exist concerning the effect of oral retinoids on bone healing, which would also be relevant to orthopedic surgeons. We did not perform an extensive review of the interaction between oral retinoids and bone healing. In short, the small pool of relevant literature we encountered suggested that women with an existing vitamin D deficiency may be at increased risk of lower bone mineral density after starting isotretinoin [52]. It is still unclear how oral retinoids impact bone healing in a clinical setting. Experimental studies suggest osteoblast cell lines decrease their expression of alkaline phosphatase and osteocalcin, markers of osteoblast maturation, when treated with retinoids. Retinoids also are associated with downregulated osteoclastogenesis in experiments [53–55]. In lab studies, osteoporotic properties such as significantly decreased bone mineral density were demonstrated in rats receiving very high doses of isotretinoin (30 mg/kg) [56]. Osteoporotic activity was not markedly observed when the dose of isotretinoin was 1 mg/kg, a dosage equivalent to human dosing, nor was osteoporotic activity significant when studying the interaction in humans. Still, Miziolek et al. recommends reducing the isotretinoin dose to 0.5 mg/kg per day or holding isotretinoin all together in the setting of a long bone fracture [53]. This recommendation is in addition to correcting any vitamin D deficiency prior to starting isotretinoin. Dermatologists should consider these findings when prescribing oral retinoids and when working collaboratively with orthopedic surgeons during a patient’s perioperative period.

### ENT/otologic surgery

Only one published paper was found on our topic as it specifically relates to otolaryngology. It differs from most of the studies we reviewed in that it evaluates how oral retinoids may play a role in the distant post-operative period, versus the perioperative studies we previously reviewed. Alwabili et al. recently shared a case report of a 25 year-old female who underwent a cartilage tympanoplasty with total ossicular replacement prosthesis (TORP) in her right ear for chronic otitis media [57]. In this procedure, a cartilage graft is placed between the prosthesis and the tympanic membrane to prevent the prosthesis from extruding [58]. Two years after the patient underwent this procedure, she was started on oral isotretinoin by her dermatologist. Previously, she had been clinically doing well post-operatively. However, after increasing her oral isotretinoin dose from 20 to 40 mg the patient noticed right-sided hearing loss and bloody otorrhea. Her otoscopic exam subsequently identified thinning of the graft and prosthesis extrusion, and the authors conclude that the oral isotretinoin use interfered with the cartilage graft and led to the complication [57]. This was the only study we found specifically relevant to ENT or head and neck surgery. Its presentation in isolation of cartilage graft failure after starting oral isotretinoin 2 years after surgery prompts the question of whether isotretinoin should be contraindicated in those who have had such otologic procedures and for how long. Given that no other data on this specific topic exists to our knowledge, we would recommend dermatologists take a thorough surgical history of patients to evaluate if they underwent procedures involving cartilage grafting. Those patients that have such procedures should be aware of this possible risk of graft thinning, and depending on the surgery, how a jeopardized cartilage graft could affect them. Additional studies are certainly needed to further characterize this potential risk.

### Maxillofacial surgery

Our review of the literature included a couple reports of patients on isotretinoin who had postoperative alveolar osteitis after having third molar surgery or other tooth extraction. The etiology of alveolar osteitis, colloquially referred to as dry socket, is believed to be due to absence or lysis of the platelet clot at the extraction site prematurely, leaving the underlying bone exposed, though the mechanism of why the platelet clot breaks down is still poorly understood [59]. A retrospective study aimed to identify if patients taking isotretinoin (or having recently discontinued isotretinoin) experienced complications after wisdom tooth extraction [60]. Of 339 adolescents and young adults prescribed isotretinoin, 26 of them had wisdom tooth extraction while taking isotretinoin or had discontinued it within the previous month. Though no patients had intraoperative complications, 3 of the 26 patients had alveolar osteitis [60]. All 3 patients were currently on isotretinoin, and all 3 patients suffered 1 dry socket each and subsequently healed without needing further intervention. Even though the percentage of patients on isotretinoin who developed alveolar osteitis in this study was greater than average cited rates (11.4% versus 3–5%), the authors concluded the small size of their study hindered them from making any association between isotretinoin use and wisdom tooth extraction postoperative complications, and there remains no evidence suggesting third molar surgery should be deferred during isotretinoin use [60]. Additionally, Tolkachjov et al.’s single-center retrospective analysis of patients exposed to isotretinoin in the perioperative period of various procedures only found wound-healing complications in the exposed group to be after dental surgery [61]. Their analysis consisted of identifying patients exposed to isotretinoin within the last 6 months and those who weren’t, and then determining what percentage of patients in each group underwent incisional surgery and had subsequent adverse events. In the exposed group, only 2 of 76 patients (2.6%) had adverse events, both of which underwent tooth extractions. One patient had alveolar osteitis and another is described as having “poor, edematous, and erythematous healing at incision sites.” [61] Compare this to the 2 of 82 (2.4%) patients in the unexposed group with reported adverse events, one of which was also a tooth extraction [61]. Again, these authors concluded their limited study and overall findings, which included all types of surgeries, challenge the current process of waiting for isotretinoin to be discontinued prior to surgery. It is interesting to consider isotretinoin in the setting of dental surgery, as the reported complication of alveolar osteitis is hypothesized to be related to platelet clot breakdown and not compromised re-epithelization, which is the manner in which isotretinoin is thought to interfere. The limited evidence presented here does not support holding oral retinoids or delaying surgery for tooth extraction.

**Table 1:** Summary of findings

Surgical Specialty	Summary of Findings
Dermatologic Surgery	Oral retinoids show no significant adverse effects on wound healing in various dermatologic procedures, according to recent literature and consensus recommendations, challenging previous beliefs regarding the need to delay procedures in patients using retinoids perioperatively.
Plastic Surgery	In cosmetic plastic surgeries, such as breast reduction surgery and chest masculinization surgery, of patients using oral retinoids, satisfactory outcomes were reported despite the risk of hypertrophic scarring, emphasizing the need for timely interventions to mitigate negative psychological effects. Perioperative use of oral retinoids in patients undergoing rhinoplasty may enhance satisfaction and cosmetic outcomes, despite potential risk of nasal tip deformities, necessitating careful consideration by plastic surgeons and dermatologists.
Ophthalmic Surgery	There is not definitive evidence that oral retinoids significantly affect outcomes of refractive surgery despite potential concerns regarding dry eye and Meibomian gland dysfunction.
Orthopedic Surgery	While the available literature lacks orthopedic-specific studies, collaboration between orthopedic surgeons and dermatologists regarding perioperative isotretinoin use should be encouraged due to potential impacts on bone healing.
ENT/Otologic Surgery	Single study suggests oral retinoids may pose risk for cartilage graft thinning in otologic surgeries.
Maxillofacial Surgery	Patients taking isotretinoin undergoing dental surgery showed cases of alveolar osteitis, but evidence doesn’t support delaying surgery or discontinuing isotretinoin preoperatively.

## Conclusion

To our knowledge, this is the first literature review to analyze isotretinoin use in the perioperative period as it relates to different surgical subspecialties. Our findings are summarized in Table 1. Little data exists outside of dermatologic surgeries and procedures, plastic and reconstructive surgery, and maxillofacial surgery. Surgical specialties not mentioned above in this paper (general surgery and its subspecialties, obstetrics and gynecology, urology, and neurosurgery) did not turn up any specialty-specific findings in our review of the current literature with regards to incisional wound healing related to perioperative oral retinoid use. However, existing data demonstrates that oral retinoids most likely do not inhibit incisional wound healing. It is not recommended to delay surgery in those currently on systemic retinoid treatment for concern of scarring or keloid formation. There may be valid concern that oral retinoids affect cartilage or muscle healing, though little to no data exists to truly draw conclusions in this regard. Further studies are needed to confidently stratify the overall risk, if any, oral retinoids pose in the perioperative period. It is difficult to conduct an interventional investigation as it would be undesirable to potentially subject research participants to an adverse aesthetic outcome. Many surgeons, understandably, are still hesitant to operate in the setting of oral retinoid use. We plan to further our investigation of this topic by surveying surgeons of varying specialties on their opinion of the subject. In conclusion, we suspect that the widely practiced concept of holding oral retinoids in the perioperative period is likely unnecessary for most incisional surgeries.

## Data Availability

No datasets were generated or analysed during the current study.

## References

[1] Ungarelli LF, Hetem CM, Farina Junior JA (2016) Is it safe to operate on patients taking isotretinoin? Aesthetic Plast Surg 40(1):139–148 26686845 10.1007/s00266-015-0588-3

[2] Ginarte M, Peteiro C, Toribio J (1999) Keloid formation induced by isotretinoin therapy. Int J Dermatol 38(3):228–229 10208624 10.1046/j.1365-4362.1999.00597.x

[3] Dogan G (2006) Possible isotretinoin-induced keloids in a patient with Behçet’s disease. Clin Exp Dermatol 31(4):535–537 16716157 10.1111/j.1365-2230.2006.02140.x

[4] Katz BE, Mac Farlane DF (1994) Atypical facial scarring after isotretinoin therapy in a patient with previous dermabrasion. J Am Acad Dermatol 30(5 Pt 2):852–853 8169260 10.1016/s0190-9622(94)70096-6

[5] Rubenstein R, Roenigk HH Jr., Stegman SJ, Hanke CW (1986) Atypical keloids after dermabrasion of patients taking isotretinoin. J Am Acad Dermatol 15(2 Pt 1):280–285 3018052 10.1016/s0190-9622(86)70167-9

[6] Zachariae H (1988) Delayed wound healing and keloid formation following argon laser treatment or dermabrasion during isotretinoin treatment. Br J Dermatol 118(5):703–706 2969261 10.1111/j.1365-2133.1988.tb02574.x

[7] Pastar I, Stojadinovic O, Yin NC, Ramirez H, Nusbaum AG, Sawaya A et al (2014) Epithelialization in Wound Healing: a Comprehensive Review. Adv Wound Care (New Rochelle) 3(7):445–464 25032064 10.1089/wound.2013.0473 PMC4086220

[8] Zouboulis CC (2006) Isotretinoin revisited: pluripotent effects on human sebaceous gland cells. J Invest Dermatol 126(10):2154–2156 16983322 10.1038/sj.jid.5700418

[9] Wootton CI, Cartwright RP, Manning P, Williams HC (2014) Should isotretinoin be stopped prior to surgery? A critically appraised topic. Br J Dermatol 170(2):239–244 24547720 10.1111/bjd.12761

[10] Waldman A, Bolotin D, Arndt KA, Dover JS, Geronemus RG, Chapas A et al (2017) ASDS guidelines Task Force: Consensus recommendations regarding the safety of lasers, Dermabrasion, Chemical peels, Energy Devices, and skin surgery during and after Isotretinoin Use. Dermatol Surg 43(10):1249–1262 28498204 10.1097/DSS.0000000000001166

[11] Hernandez-Perez E, Khawaja HA, Alvarez TYM (2000) Oral isotretinoin as part of the treatment of cutaneous aging. Dermatol Surg. 2000;26(7):649–652 10.1046/j.1524-4725.2000.99210.x 10886272

[12] Kar BR, Tripathy S, Panda M (2013) Comparative study of oral isotretinoin versus oral isotretinoin + 20% salicylic acid peel in the treatment of active acne. J Cutan Aesthet Surg 6(4):204–208 24470716 10.4103/0974-2077.123403 PMC3884884

[13] Roenigk HH Jr., Pinski JB, Robinson JK, Hanke CW (1985) Acne, retinoids, and dermabrasion. J Dermatol Surg Oncol 11(4):396–398 3156902 10.1111/j.1524-4725.1985.tb01291.x

[14] Bagatin E, dos Santos Guadanhim LR, Yarak S, Kamamoto CS, de Almeida FA (2010) Dermabrasion for acne scars during treatment with oral isotretinoin. Dermatol Surg 36(4):483–489 20180836 10.1111/j.1524-4725.2010.01474.x

[15] Picosse FR, Yarak S, Cabral NC, Bagatin E (2012) Early chemabrasion for acne scars after treatment with oral isotretinoin. Dermatol Surg 38(9):1521–1526 22687256 10.1111/j.1524-4725.2012.02460.x

[16] Plewig G, Nikolowski J, Wolff HH (1982) Action of isotretinoin in acne rosacea and gram-negative folliculitis. J Am Acad Dermatol 6(4 Pt 2 Suppl):766–785 6461680 10.1016/s0190-9622(82)70067-2

[17] Mirza FN, Mirza HN, Khatri KA (2020) Concomitant use of isotretinoin and lasers with implications for future guidelines: an updated systematic review. Dermatol Ther 33(6):e14022 32677092 10.1111/dth.14022

[18] Bernestein LJ, Geronemus RG (1997) Keloid formation with the 585-nm pulsed dye laser during isotretinoin treatment. Arch Dermatol 133(1):111–112 9006390 10.1001/archderm.1997.03890370123029

[19] Mandrell J, Youker S, Allen EJ, Hurley MY, Obadiah J (2010) Keloid formation occurring in the distribution of a congenital vascular malformation. Skinmed. 8(5):298–300 21137643

[20] Noishiki C, Hayasaka Y, Ogawa R (2019) Sex differences in Keloidogenesis: an analysis of 1659 keloid patients in Japan. Dermatol Ther (Heidelb) 9(4):747–754 31586308 10.1007/s13555-019-00327-0 PMC6828900

[21] Michael AI, Ademola SA, Olawoye OA, Iyun AO, Adebayo W, Oluwatosin OM (2017) Pediatric keloids: a 6-year retrospective review. Pediatr Dermatol 34(6):673–676 29023993 10.1111/pde.13302

[22] Davis SA, Feldman SR, McMichael AJ (2013) Management of keloids in the United States, 1990–2009: an analysis of the National Ambulatory Medical Care Survey. Dermatol Surg 39(7):988–994 23463963 10.1111/dsu.12182

[23] Ogawa R, Okai K, Tokumura F, Mori K, Ohmori Y, Huang C et al (2012 Mar-Apr) The relationship between skin stretching/contraction and pathologic scarring: the important role of mechanical forces in keloid generation. Wound repair Regen. 20(2):149–157 10.1111/j.1524-475X.2012.00766.x 22332721

[24] Chandrashekar BS, Varsha DV, Vasanth V, Jagadish P, Madura C, Rajashekar ML (2014) Safety of performing invasive acne scar treatment and laser hair removal in patients on oral isotretinoin: a retrospective study of 110 patients. Int J Dermatol 53(10):1281–1285 25039864 10.1111/ijd.12544

[25] Yoon JH, Park EJ, Kwon IH, Kim CW, Lee GS, Hann SK et al (2014) Concomitant use of an infrared fractional laser with low-dose isotretinoin for the treatment of acne and acne scars. J Dermatolog Treat 25(2):142–146 23336106 10.3109/09546634.2013.768758

[26] Leal H, MD, UltraLaser, Monterrey M, Priscila Cantu MD, UltraLaser (2011) Monterrey, Mexico. Fractionated erbium laser during oral isotretinoin treatment. J Am Acad Dermatol 64(2):AB18

[27] Kingsley MG (2012) Bae-Harboe, Yoon Soo Cindy. Treatment of acne scarring with ablative fractionated laser resurfacing following isotretinoin therapy. J Am Acad Dermatol 66(4):AB216

[28] Kim HW, Chang SE, Kim JE, Ko JY, Ro YS (2014) The safe delivery of fractional ablative carbon dioxide laser treatment for acne scars in Asian patients receiving oral isotretinoin. Dermatol Surg 40(12):1361–1366 25361201 10.1097/DSS.0000000000000185

[29] Park HC, Kim EJ, Kim JE, Ro YS, Ko JY (2013) Treatment of multiple eccrine hidrocystomas with isotretinoin followed by carbon dioxide laser. J Dermatol 40(5):414–415 23414089 10.1111/1346-8138.12059

[30] Khatri KA (2004) Diode laser hair removal in patients undergoing isotretinoin therapy. Dermatol Surg 30(9):1205–1207 discussion 7 15355360 10.1111/j.1524-4725.2004.30373.x

[31] Cassano N, Arpaia N, Vena GA (2005) Diode laser hair removal and isotretinoin therapy. Dermatol Surg 31(3):380–381 15841649 10.1111/j.1524-4725.2005.31097_1

[32] Khatri KA, Garcia V (2006) Light-assisted hair removal in patients undergoing isotretinoin therapy. Dermatol Surg 32(6):875–877 16792663 10.1111/j.1524-4725.2006.32182.x

[33] Khatri KA (2009) The safety of long-pulsed nd:YAG laser hair removal in skin types III-V patients during concomitant isotretinoin therapy. J Cosmet Laser Ther 11(1):56–60 19199117 10.1080/14764170802612984

[34] Patwardhan MM, Bijal; Kangle S, Singh S (2012) Isotretinoin and lasers: can they be used at the same time? J Am Acad Dermatol 66(4):AB28

[35] Patwardhan Andrade M (2016) Isotretinoin and lasers: friends or foes. J Am Acad Dermatol 74(5):AB19

[36] Ibrahim SM, Farag A, Hegazy R, Mongy M, Shalaby S, Kamel MM (2021) Combined low-dose isotretinoin and pulsed Dye Laser Versus standard-dose isotretinoin in the treatment of inflammatory acne. Lasers Surg Med 53(5):603–609 33185932 10.1002/lsm.23356

[37] Yew YW, Pan JY (2014) Complete remission of recalcitrant genital warts with a combination approach of surgical debulking and oral isotretinoin in a patient with systemic lupus erythematosus. Dermatol Ther 27(2):79–82 10.1111/dth.12059 24703263

[38] Larson DL, Flugstad NA, O’Connor E, Kluesner KA, Plaza JA (2012) Does systemic isotretinoin inhibit healing in a porcine wound model? Aesthet Surg J 32(8):989–998 23110930 10.1177/1090820X12462383

[39] Tan SR, Tope WD (2004) Effect of acitretin on wound healing in organ transplant recipients. Dermatol Surg 30(4 Pt 2):667–673 15061853 10.1111/j.1524-4725.2004.00154.x

[40] ASPS (2020) Plastic Surgery Statistics Report. 2020

[41] Davies MJ, Perkins D (2022) Oral isotretinoin (roaccutane) use during incisional surgery: safe or risky? Case Rep Plast Surg Hand Surg 9(1):131–134 10.1080/23320885.2022.2071275 PMC9116253 35601983

[42] Strock D, Sivesind TE, Dellavalle RP, Mundinger GS (2023) Isotretinoin Use in Transmasculine patients and its implication on chest masculinization surgery: scoping review of the literature. JMIR Dermatol 6:e45351 37616418 10.2196/45351 PMC10450534

[43] Kaymak Y (2008) Creatine phosphokinase values during isotretinoin treatment for acne. Int J Dermatol 47(4):398–401 18377609 10.1111/j.1365-4632.2008.03491.x

[44] Allen BC, Rhee JS (2005) Complications associated with isotretinoin use after rhinoplasty. Aesthetic Plast Surg 29(2):102–106 10.1007/s00266-004-0041-5 15803349

[45] Yahyavi S, Jahandideh H, Izadi M, Paknejad H, Kordbache N, Taherzade S (2020) Analysis of the effects of Isotretinoin on Rhinoplasty patients. Aesthet Surg J 40(12):Np657–np65 32756944 10.1093/asj/sjaa219

[46] Sazgar AA, Majlesi A, Shooshtari S, Sadeghi M, Sazgar AK, Amali A (2019) Oral isotretinoin in the Treatment of Postoperative Edema in Thick-Skinned Rhinoplasty: a randomized placebo-controlled clinical trial. Aesthetic Plast Surg 43(1):189–195 30288563 10.1007/s00266-018-1252-5

[47] Fraunfelder FT, Fraunfelder FW, Edwards R (2001) Ocular side effects possibly associated with isotretinoin usage. Am J Ophthalmol 132(3):299–305 11530040 10.1016/s0002-9394(01)01024-8

[48] Ortega-Usobiaga J, Rocha-de-Lossada C, Llovet-Rausell A, Llovet-Osuna F (2023) Update on contraindications in laser corneal refractive surgery. Archivos De La Sociedad Española De Oftalmología (English Edition). 98(2):105–111 10.1016/j.oftale.2022.07.003 36114139

[49] Toda I, Dry Eye After LASIK (2018) Investig Ophthalmol Vis Sci 59(14):DES109–DES15 30481814 10.1167/iovs.17-23538

[50] Ortega-Usobiaga J, Llovet-Osuna F, Djodeyre MR, Bilbao-Calabuig R, González-López F, Llovet-Rausell A et al (2018) Outcomes of laser in situ keratomileusis and Photorefractive Keratectomy in patients taking isotretinoin. Am J Ophthalmol 192:98–103 29772222 10.1016/j.ajo.2018.05.009

[51] Spierer O, Nemet A, Bloch S, Israeli A, Mimouni M, Kaiserman I (2023) The Effect of Meibomian Gland Dysfunction on laser-assisted in situ keratomileusis in asymptomatic patients. Ophthalmol Ther 12(1):281–291 36348201 10.1007/s40123-022-00610-y PMC9834456

[52] Saadi H, Afandi B, Houssami L, Saleh N, Mohamadiyeh M, Benedict S (2019) Effects of isotretinoin on bone turnover markers and bone mineral density in women with acne vulgaris and vitamin D deficiency: a preliminary study. Int J Diabetes Metabolism 16(3):107–112

[53] Miziołek B, Bergler-Czop B, Stańkowska A, Brzezińska-Wcisło L (2019) The safety of isotretinoin treatment in patients with bone fractures. Postepy Dermatol Alergol 36(1):18–24 30858774 10.5114/ada.2019.82822 PMC6409881

[54] Lind T, Sundqvist A, Hu L, Pejler G, Andersson G, Jacobson A et al (2013) Vitamin a is a negative regulator of osteoblast mineralization. PLoS ONE 8(12):e82388 24340023 10.1371/journal.pone.0082388 PMC3858291

[55] Green AC, Poulton IJ, Vrahnas C, Häusler KD, Walkley CR, Wu JY et al (2015) RARγ is a negative regulator of osteoclastogenesis. J Steroid Biochem Mol Biol 150:46–53 25800721 10.1016/j.jsbmb.2015.03.005

[56] Hotchkiss CE, Latendresse J, Ferguson SA (2006) Oral treatment with retinoic acid decreases bone mass in rats. Comp Med 56(6):502–511 17219781

[57] Alwabili M, Alotaibi N, Alamry S (2022) Prosthesis extrusion post total ossicular replacement ossiculoplasty (TORP) following isotretinoin use: a case report and literature review of peri-operative isotretinoin safety. Ann Med Surg (Lond) 81:104469 36147053 10.1016/j.amsu.2022.104469 PMC9486709

[58] Saliba I, Sabbah V, Poirier JB (2018) Total ossicular replacement prosthesis: a New Fat Interposition technique. Clin Med Insights Ear Nose Throat 11:1179550617749614 29326537 10.1177/1179550617749614 PMC5757430

[59] Rohe C, Schlam M (2024) Alveolar osteitis. StatPearls. Treasure Island (FL) ineligible companies. Disclosure: Mark Schlam declares no relevant financial relationships with ineligible companies.: StatPearls Publishing Copyright © 2024. StatPearls Publishing LLC.

[60] Sharma J, Thiboutot DM, Zaenglein AL (2012) The effects of isotretinoin on wisdom tooth extraction. J Am Acad Dermatol 67(4):794–795 22980255 10.1016/j.jaad.2011.02.001

[61] Tolkachjov SN, Sahoo A, Patel NG, Lohse CM, Murray JA, Tollefson MM (2017) Surgical outcomes of patients on isotretinoin in the perioperative period: a single-center, retrospective analysis. J Am Acad Dermatol 77(1):159–161 28619552 10.1016/j.jaad.2017.03.019

